# Impact of poly(A)-tail G-content on *Arabidopsis* PAB binding and their role in enhancing translational efficiency

**DOI:** 10.1186/s13059-019-1799-8

**Published:** 2019-09-03

**Authors:** Taolan Zhao, Qing Huan, Jing Sun, Chunyan Liu, Xiuli Hou, Xiang Yu, Ian M. Silverman, Yi Zhang, Brian D. Gregory, Chun-Ming Liu, Wenfeng Qian, Xiaofeng Cao

**Affiliations:** 1 State Key Laboratory of Plant Genomics and National Center for Plant Gene Research, Institute of Genetics and Developmental Biology, Chinese Academy of Sciences, Beijing, 100101 China; 2 Key Laboratory of Genetic Network Biology, Institute of Genetics and Developmental Biology, Chinese Academy of Sciences, Beijing, 100101 China; 3 University of Chinese Academy of Sciences, Beijing, 100049 China; 4 Key Laboratory of Plant Molecular Physiology, Institute of Botany, Chinese Academy of Sciences, Beijing, 100093 China; 5 Department of Biology, University of Pennsylvania, Philadelphia, PA 19104 USA; 6 Laboratory for Genome Regulation and Human Health and Center for Genome Analysis, ABLife Inc, Wuhan, 430075 Hubei China; 7 CAS Center for Excellence in Molecular Plant Sciences, Institute of Genetics and Developmental Biology, Chinese Academy of Sciences, Beijing, 100101 China

**Keywords:** Poly(A) tails, Poly(A)-binding proteins, PAB binding efficiency, Poly(A)-tail G-content, mRNA stability, Translational efficiency, *Arabidopsis*

## Abstract

**Background:**

Polyadenylation plays a key role in producing mature mRNAs in eukaryotes. It is widely believed that the poly(A)-binding proteins (PABs) uniformly bind to poly(A)-tailed mRNAs, regulating their stability and translational efficiency.

**Results:**

We observe that the homozygous triple mutant of broadly expressed *Arabidopsis thaliana* PABs, AtPAB2, AtPAB4, and AtPAB8, is embryonic lethal. To understand the molecular basis, we characterize the RNA-binding landscape of these PABs. The AtPAB-binding efficiency varies over one order of magnitude among genes. To identify the sequences accounting for the variation, we perform poly(A)-seq that directly sequences the full-length poly(A) tails. More than 10% of poly(A) tails contain at least one guanosine (G); among them, the G-content varies from 0.8 to 28%. These guanosines frequently divide poly(A) tails into interspersed A-tracts and therefore cause the variation in the AtPAB-binding efficiency among genes. Ribo-seq and genome-wide RNA stability assays show that AtPAB-binding efficiency of a gene is positively correlated with translational efficiency rather than mRNA stability. Consistently, genes with stronger AtPAB binding exhibit a greater reduction in translational efficiency when AtPAB is depleted.

**Conclusions:**

Our study provides a new mechanism that translational efficiency of a gene can be regulated through the G-content-dependent PAB binding, paving the way for a better understanding of poly(A) tail-associated regulation of gene expression.

**Electronic supplementary material:**

The online version of this article (10.1186/s13059-019-1799-8) contains supplementary material, which is available to authorized users.

## Background

Various RNA-binding proteins regulate almost every step of an mRNA’s life, from birth (transcription) to death (degradation) [1, 2]. A conserved family of RNA-binding proteins, the cytoplasmic poly(A)-binding proteins (PABs) [3, 4], was first purified from mammalian cells as proteins covering the poly(A) tails of mRNAs [5, 6]. PABs are encoded by a single gene in the budding yeast (*Saccharomyces cerevisiae*) and fruit flies (*Drosophila melanogaster*) and by duplicate genes in *Caenorhabditis elegans*, *Xenopus*, mice, and humans [7]. Eight *Arabidopsis thaliana PAB* genes have been identified based on sequence similarity [8]. Three of them, *AtPAB2*, *AtPAB4*, and *AtPAB8*, are highly expressed in a wide range of tissues and developmental stages [8]. The double mutants *atpab2 atpab4* and *atpab2 atpab8* exhibited pleiotropic developmental abnormalities in leaf shape, silique growth, plant height, and flowering time [9, 10]. They also displayed reduced ethylene sensitivity [11], enhanced *Turnip mosaic virus* resistance [9], and defects in pattern-triggered immunity [12]. These observations indicate a key role of AtPABs in basic cellular functions.

It is generally assumed that PABs uniformly bind poly(A)-tailed mRNAs [6, 13]. Based on this idea, a method was developed to isolate tissue-specific mRNA; the epitope-tagged PAB was expressed from a tissue-specific promoter and was used to indiscriminately immunoprecipitate mRNA in the tissue [14, 15]. Intriguingly, recent studies discovered the integration of non-A nucleotides (C, G, or U) into the poly(A) tail [16–19]. In HeLa cells, guanosine was identified as the most abundant non-A nucleotide in poly(A) tails and exhibited a variable frequency among genes [16, 19]. These non-A nucleotides potentially affect PAB binding since the RNA recognition motifs of PABs mainly bind to A-tracts [20–22]. For example, a stretch of 11 or 12 consecutive A’s is required for human PAB or yeast Pab1p binding, respectively [20, 23].

In spite of the importance of PABs and their ubiquitous binding to mRNA poly(A) tails, the molecular function of PAB binding remains unclear. Although the regulatory roles of PABs in mRNA stability and translational efficiency have been reported, most evidence was from cell-free systems and reporter assays [3, 24, 25]. For example, depletion of PABs in cell extracts promoted the degradation of poly-adenylated β-globin mRNAs, while refilling PABs to the system re-stabilized those reporter mRNAs [26]. In addition, PABs facilitated the recruitment of the 40S ribosomal subunit [27] as well as the assembly of the 80S ribosome initiation complex in an in vitro translation system [28, 29]. Moreover, PABs interacted with eukaryotic translation initiation factor 4G (eIF4G) to synergistically promote the translation of luciferase reporters [30–32]. Collectively, the regulatory roles of PABs on the expression of endogenous genes remain largely unknown.

## Results

### AtPABs are essential in plants

**Fig. 1:**
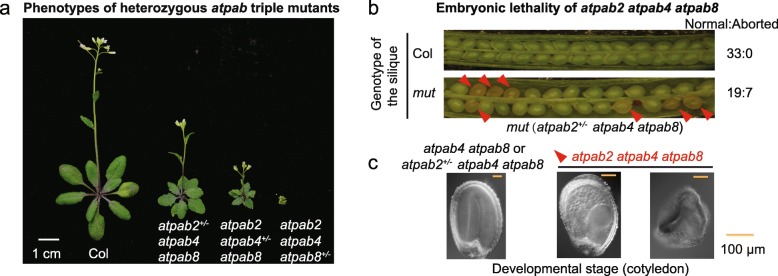
AtPABs play essential roles in plants. **a** Heterozygous *atpab* triple mutants exhibited various phenotypic abnormalities, such as dwarf, multi-branches, premature senility, serrated leaves, and sterility. **b** About 25% aborted ovules (indicated by red triangles) were observed in the siliques of the *mut* (*atpab2*^*+/−*^ *atpab4 atpab8*). **c** Embryos of *atpab2 atpab4 atpab8* degenerated when normal embryos reached the cotyledon stage. Embryos were obtained from a single silique of the *mut*

*AtPAB2*, *AtPAB4*, and *AtPAB8* are highly and consistently expressed (Additional file 1: Figure S1). To study the biological functions of them, we constructed their mutants (Fig. 1a; Additional file 1: Figure S2a, b). Triple mutants where one gene was heterozygous (*atpab2*^+/−^ *atpab4 atpab8*, *atpab2 atpab4*^+/−^ *atpab8*, and *atpab2 atpab4 atpab8*^+/−^) were obtained and exhibited various levels of phenotypic abnormality (Fig. 1a), although the single mutants did not [9], implying functional redundancy among *AtPAB2*, *AtPAB4*, and *AtPAB8*. The *atpab2*^+/−^ *atpab4 atpab8* mutant (refer to as *mut* hereafter) was used for further analysis since the other two heterozygous mutants barely flowered.

The homozygous triple mutant *atpab2 atpab4 atpab8* was embryonic lethal, as supported by the following four lines of evidence. First, we failed to generate the homozygous triple mutant from the self-cross of the *mut*. Second, a self-cross of the *mut* plants produced *atpab2*^+/+^ *atpab4 atpab8* and *mut* progeny in a 1:2 ratio (*P* = 0.56, the binomial test, Additional file 2: Table S1). Third, ~ 25% of seeds were aborted in the *mut* siliques (*P* = 0.33, Fig. 1b; Additional file 2: Table S2). Fourth, we observed the asynchronous development of embryos within the same *mut* silique (Additional file 2: Tables S3 and Table S4); at the time normal embryos reached the walking stick stage, ~ 25% of embryos in *mut* siliques were arrested at the torpedo stage and later degenerated (Fig. 1c; Additional file 1: Figure S2c; Additional file 2: Table S4).

To understand why homozygous triple mutant was embryonic lethal, we detected the expression of AtPAB2, AtPAB4, and AtPAB8 during embryo development. We constructed reporters by fusing each AtPAB with the green fluorescent protein (GFP) and hemagglutinin (HA) and expressed the fusion proteins from their respective native promoters (Additional file 1: Figure S3a, b). The fusion proteins (AtPAB2-GFP-HA, AtPAB4-GFP-HA, and AtPAB8-GFP-HA) rescued the phenotypes of *atpab* double mutants (Additional file 1: Figure S3c, d), indicating that they retained AtPAB function. Using these complementation lines, we observed universally and constitutively cytoplasmic expression patterns of AtPABs during seed generation (Additional file 1: Figure S4). These observations indicate that AtPABs play ubiquitous roles in basic cellular functions.

### mRNAs bind AtPABs with various efficiencies

To understand how AtPABs function in a cell, we investigated the RNA-binding profiles of AtPABs. We performed electrophoretic mobility shift assays (EMSA), in which unlabeled RNA fragments competed with the radio-labeled ones for protein binding so that the direct interaction between protein and RNA was detected. We observed a strong binding of these three AtPABs to radio-labeled oligo(A)_30_ (Additional file 1: Figure S5a), indicating AtPABs as functional poly(A)-binding proteins.

**Fig. 2:**
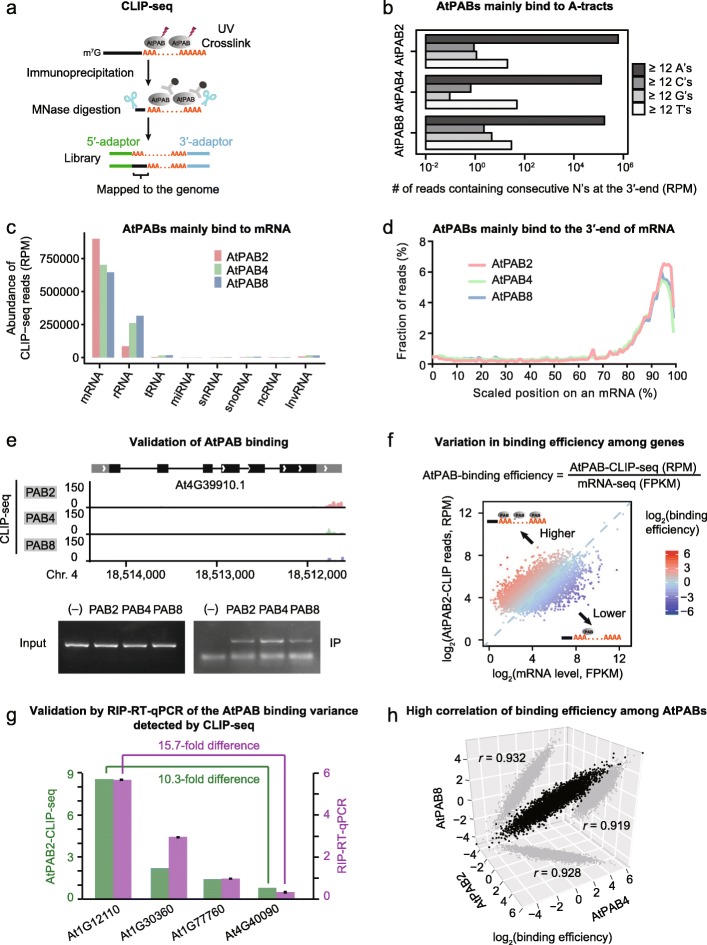
mRNAs bind to AtPABs with different efficiencies. **a** Schematic of the CLIP-seq experiment, which detects the direct RNA-binding targets of AtPABs. **b** AtPABs bind predominantly to consecutive A’s. Reads with ≥ 12 consecutive N’s were counted because the yeast Pab1p requires at least 12 consecutive A’s for binding. **c** mRNA is the major binding target of AtPABs. **d** CLIP-seq reads were mainly mapped to the 3′-ends of mRNAs. The length of each mRNA was scaled to 100%. “0” and “100” represent the transcription start and end, respectively. **e** The AtPAB-binding gene detected in CLIP-seq was validated by RIP-RT-PCR. The distribution of the AtPAB-CLIP reads is shown by the wiggle plots. The gene model shows the untranslated regions (gray boxes), coding sequences (black boxes), and introns (lines). The input and IP panels show the mRNA level of an AtPAB-binding gene in total RNA and RIP experiments (anti-HA antibody), respectively. The minus sign (−) indicates the negative control (the wild-type Col) in which the GFP-HA tagged AtPAB is absent. **f** The AtPAB2-binding efficiency significantly varies among genes in Col. Each dot represents a target gene of AtPAB2. The diagonal is shown by a blue dashed line. **g** The difference in AtPAB2-binding efficiency among genes was validated by RIP-RT-qPCR. Green bars and purple bars show the AtPAB2-binding efficiency estimated by CLIP-seq and RIP-RT-qPCR, respectively. The ~ 10-fold difference in AtPAB2-binding efficiency between At1G12110 and At4G40090 that was detected by CLIP-seq was validated by RIP-RT-qPCR. The error bar represents the standard deviation of three replicates. **h** The binding efficiencies of genes were highly correlated among AtPABs. *P* values were given by Pearson’s correlation analysis (*P* < 1 × 10^−100^ in all three pairwise comparisons)

We further characterized the RNA-binding landscape of AtPABs in vivo. We performed protein-RNA crosslinking and immunoprecipitation (CLIP)-seq [33] to identify the direct RNA-binding targets of AtPABs (Fig. 2a; Additional file 1: Figure S5b-e). More than 15 million raw reads were obtained in each CLIP library (Additional file 2: Table S5). Sequencing reads containing consecutive A’s dominated the AtPAB-CLIP libraries (Fig. 2b), suggesting that AtPAB-binding RNAs were successfully captured. To identify transcripts binding to AtPABs, we removed consecutive A’s from the 3′-end of each read and mapped the remaining sequence to the *Arabidopsis* genome (Fig. 2a). Mapped reads were enriched at the 3′-terminus of mRNAs (Fig. 2c, d), indicating that the non-poly(A) sequences in the AtPAB-CLIP reads likely hitchhiked on poly(A) tails during AtPAB-CLIP. Therefore, mRNA poly(A) tails are the major targets of AtPABs in plant cells.

To identify transcripts that are bound by AtPABs, we clustered uniquely mapped reads into binding sites using Pyicoclip [34]. 8857, 7311, and 6300 binding clusters were identified in 8322, 6292, and 5356 genes, for AtPAB2, AtPAB4, and AtPAB8, respectively (Additional file 2: Table S5). To validate the binding genes detected by CLIP-seq, we randomly chose ten genes and successfully confirmed their binding with AtPABs by RNA immunoprecipitation coupled reverse transcription and polymerase chain reaction (RIP-RT-PCR; one example in Fig. 2e; others in Additional file 1: Figure S6). The global nature of the RNA-binding targets of AtPABs echoes the essential role of AtPABs in basic cellular functions (Fig. 1).

PABs were generally considered to uniformly bind poly(A)-tailed mRNAs [6, 13, 15]. To determine if this is true, we plotted the read abundance of the genes in the AtPAB-CLIP against mRNA levels (Fig. 2f; Additional file 1: Figure S7). If all mRNAs are bound uniformly, we would expect them on a diagonal line; however, we observed a substantial deviation from the diagonal (Fig. 2f; Additional file 1: Figure S7), indicating that mRNAs vary in their propensity for AtPAB binding. To assess the variation quantitatively, we defined the AtPAB-binding efficiency for each mRNA as the abundance of reads mapped to the gene in the AtPAB-CLIP normalized to its mRNA level (Fig. 2f). A large variation was observed among genes (Fig. 2f; Additional file 1: Figure S7) and notably, a > 10-fold difference in binding efficiency detected by CLIP-seq was validated with RIP-RT-coupled quantitative PCR (RIP-RT-qPCR; Fig. 2g).

The mRNA-binding landscape was highly correlated among AtPAB2, AtPAB4, and AtPAB8 (*r* > 0.9 in all three pairwise comparisons, Pearson’s correlation, Fig. 2h), indicating their largely undifferentiated roles in binding various mRNAs. This observation, echoing the absence of growth defects in the *atpab* single mutants in spite of the lethal phenotype of the triple mutant (Fig. 1), implies the functional redundancy among AtPAB2, AtPAB4, and AtPAB8.

### G-content in the poly(A) tail contributes to the variance in AtPAB-binding efficiency among genes

What causes the variation in the AtPAB-binding efficiency among genes? Considering the non-A nucleosides (especially guanosine) discovered in human poly(A) tails [19] and the A-tracts (or AU-tracts) binding preference of PABs [20, 22, 23], we propose a hypothesis that AtPAB binding is inhibited by G-residues in the poly(A) tail.

**Fig. 3:**
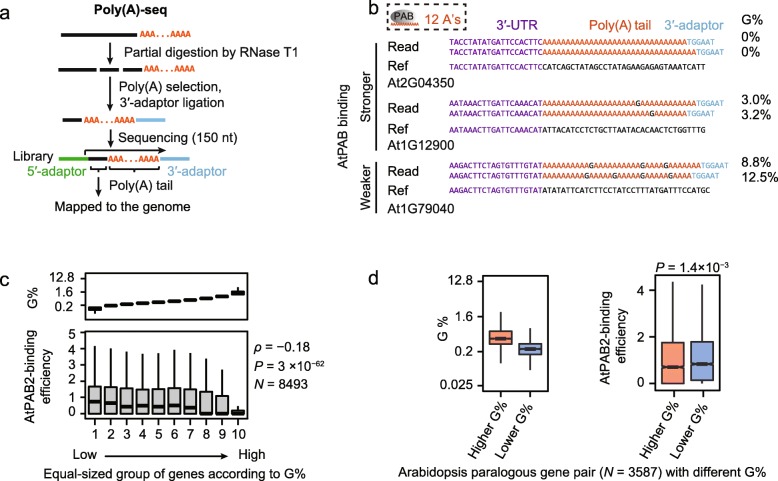
G-content contributes to the binding variance of AtPABs. **a** Schematic of the poly(A)-seq experiment that directly sequences the poly(A) tails of mRNAs. High-quality reads with recognizable Illumina 3′-adaptor sequences were used for further analysis. **b** Example reads from the poly(A)-seq show different propensities for AtPAB binding among genes. Yeast Pab1p and human PABP require at least 12 and 11 consecutive A’s for binding, respectively. Guanosines can inhibit AtPAB binding by cutting the poly(A) tail into fragments with < 12 consecutive A’s. Ref represents the reference *Arabidopsis* genome. **c** The G% was negatively correlated with the AtPAB-binding efficiency of a gene. Genes were divided into ten equal-size groups according to the average G% in the poly(A) tails. *P* values were given by the Spearman’s correlation. **d** Paralogous genes with lower G% in the poly(A) tail exhibited higher AtPAB2-binding efficiencies than their within-species paralogs with higher G% in the poly(A) tails. *P* values were given by the paired Mann-Whitney *U* test

To test this hypothesis, we developed an approach that directly sequenced the full-length poly(A) tails (poly(A)-seq) in *Arabidopsis*, by ligating RNA adaptors to the 3′-ends of poly(A) tails (Fig. 3a; Additional file 2: Table S6). To avoid the low-quality reads caused by polymerase slippage on the mononucleotide A-runs, we estimated the G-content (G%) using the reads of which the 3′-adaptor was recognizable at the 3′-terminus (Fig. 3a). In these reads, 15.8% of poly(A)-tails contained at least one non-A residue, among which guanosine had the highest proportion; 11.5% of poly(A)-tails and 34.2% of genes contained at least one guanosine, with G% varying from 0.8 to 28% among transcripts (Fig. 3b; Additional file 1: Figure S8a). These guanosines frequently cut the poly(A) tails into fragments with < 12 consecutive A’s (Fig. 3b). Consistently, the average G% in the poly(A) tails of an mRNA was negatively correlated with its AtPAB-binding efficiency among *Arabidopsis* genes (*ρ* = − 0.18, *P* = 3 × 10^−62^, Spearman’s correlation, AtPAB2 in Fig. 3c; AtPAB4 and AtPAB8 in Additional file 1: Figure S8b). Collectively, these observations suggest that the in vivo poly(A)-binding landscape of AtPABs depends on G% in the poly(A) tail.

Three lines of evidence indicate that this observation cannot result from sequencing errors. First, the filtered G-containing poly(A) tails had an average Phred quality score ~ 35, meaning ~ 3 sequencing errors per 10,000 sites. This error rate is much lower than the observed G%. Second, among these reads, the quality score of G-sites was significantly greater than that of 3′-adaptors (*P* < 10^−100^, Mann-Whitney *U* test); the validity of the latter can be confirmed by the pre-knowledge of the adaptor sequence. Third, we removed the reads where any G has a quality score smaller than 20 and still observed the G%-dependent poly(A)-binding landscape (Additional file 1: Figure S8c).

To determine whether a change in G% during evolution can cause a difference in the AtPAB-binding efficiency, we identified 3587 paralogous gene pairs in *Arabidopsis* with different G% in their poly(A) tails. The AtPAB-binding efficiency increased as G% decreased (*P* = 0.001, the paired Mann-Whitney *U* test, AtPAB2 in Fig. 3d; AtPAB4 and AtPAB8 in Additional file 1: Figure S8d), indicating the coevolution between G% and the AtPAB-binding efficiency.

### AtPAB binding enhances translational efficiency

**Fig. 4:**
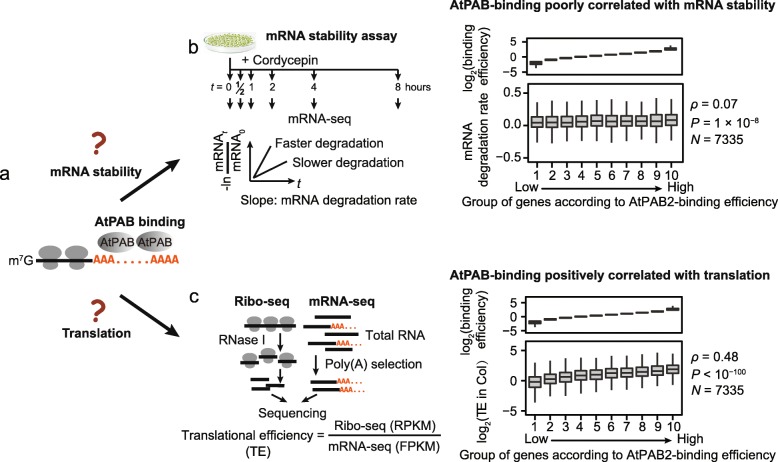
AtPAB-binding efficiency positively correlates with translational efficiency. **a** The possible consequences of AtPAB binding to mRNAs. **b** The AtPAB-binding efficiency was poorly correlated with the mRNA degradation rate in Col. **c** The AtPAB-binding efficiency was positively correlated with the translational efficiency (TE) in Col

Does AtPAB binding affect mRNA stability and translational efficiency [7]? To address these questions (Fig. 4a), we estimated mRNA stability and translational efficiency in the 2-week-old seedlings of the wild-type Col and calculated their respective correlations with the AtPAB-binding efficiency among genes. We estimated the stability of an mRNA from the rate of change in abundance throughout the time course after the transcriptional inhibitor cordycepin was added (Fig. 4b; Additional file 2: Tables S7 and S8) [35, 36]. We estimated translational efficiency using ribosome profiling (ribo-seq) that sequenced ribosome-protected fragments [37, 38]. Reads from ribo-seq showed a clear 3-nucleotide periodicity (Additional file 1: Figure S9a), indicating that the ribosome-protected fragments were successfully captured. After normalized by its mRNA level, the abundance of ribosome-protected fragments reflected the translational efficiency of a gene (Fig. 4c; Additional file 2: Tables S8, S9, and S10). We found that the AtPAB-binding efficiency was marginally correlated with mRNA degradation rate (*ρ* = 0.07, *P* = 1 × 10^−8^, AtPAB2 in Fig. 4b; AtPAB4 and AtPAB8 in Additional file 1: Figure S9b, c) but was positively correlated with translational efficiency (*ρ* = 0.48, *P* < 10^−100^, Fig. 4c; AtPAB4 and AtPAB8 in Additional file 1: Figure S9d, e), suggesting the major consequence of PAB binding in translational enhancement.

Considering that G% is a determinant of AtPAB-binding efficiency (Fig. 3c, d), we ask if G% in the poly(A) tail contributes to the variation in translational efficiency among genes. To this end, we built linear models and used Akaike information criterion (AIC) to estimate the relative quality of models; a lower AIC value represents a better model. We found that integrating G% into the linear model predicting translational efficiency significantly reduced AIC (Table 1, model 1 vs. model 2). Genome-wide associations are sometimes confounded by other factors; therefore, we built a set of linear models that include the mRNA level and/or the poly(A) length as covariants. Including G% into the models reduced AIC in all scenarios (Table 1, models 3–8), indicating the inhibitory role of guanosines in the regulation of translation efficiency, likely through AtPAB binding.

**Table 1:** Models on features that predict translational efficiency (TE)

	Model	AIC
1	Null model	15,773
2	TE ~ G%	15,770
3	TE ~ mRNA level^1^	15,031
4	TE ~ mRNA level + G%	14,968
5	TE ~ poly(A)-tail length^2^	15,773
6	TE ~ poly(A)-tail length + G%	15,769
7	TE ~ mRNA level + poly(A)-tail length	15,033
8	TE ~ mRNA level + poly(A)-tail length + G%	14,968

^1^The mRNA level of each gene in Col

^2^The median poly(A)-tail length of all transcripts of a gene in Col

**Fig. 5:**
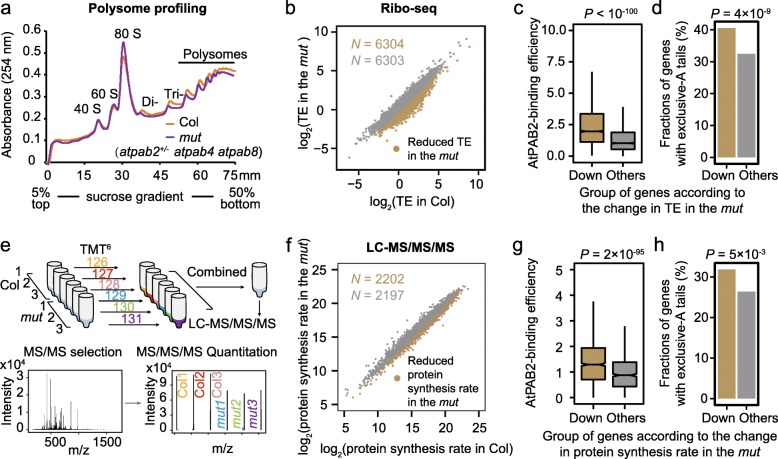
AtPABs enhance translational efficiency. **a** Polysome profiling shows the global reduction in TE in the *mut*. The *x*-axis indicates the detecting distance from 0 to 75 mm of the 5–50% sucrose gradient. **b**, **c** Genes showing reduced TE in the *mut* (**b**) exhibited significantly higher AtPAB2-binding efficiencies (**c**). The TE was calculated with the ribo-seq data. Genes with decreased TE in the *mut* (brown dots) were defined as those with TE fold change smaller than the median. *P* value in **c** was given by the Mann-Whitney *U* test. **d** Genes with exclusive adenosines in the poly(A) tail tended to be present in the gene group showing the downregulation of TE in the *mut* (defined in **b**). **e** Schematic of the TMT (Tandem Mass Tag)-based quantitative proteomics analysis. Protein samples extracted from three biological replicates of Col and the *mut* were labeled with TMT 6 plex reagent separately. **f**–**h** The protein synthesis rate was calculated as the protein level of a gene normalized to its mRNA level, and similar results were obtained as those in **b**–**d**

To further test the role of AtPABs in enhancing translation, we compared polysome profiles between the wild-type Col and the *mut* seedlings at the 2-week-old stage. The latter exhibited a reduced abundance of polysomes (Fig. 5a; Additional file 1: Figure S10a). We also performed polysome profiling using the 6-week-old plants of *atpab2 atpab4* (Additional file 1: Figure S10b), which displayed more severe developmental defects; consistently, a greater decrease in polysome abundance was observed (Additional file 1: Figure S10b). Both observations suggest a global reduction in translational efficiency when AtPABs are in short supply. Furthermore, we performed ribo-seq in the *mut* and identified 6304 genes with a reduction in translational efficiency (Fig. 5b). These genes exhibited significantly higher AtPAB-binding efficiencies (*P* < 10^−100^, Fig. 5c; Additional file 1: Figure S10c) and higher fractions of exclusive-A tails (Fig. 5d, *P* = 4 × 10^−9^, Fisher’s exact test), indicating that AtPABs likely promote translation through the binding to the poly(A) tails of the respective genes.

To determine if the difference in AtPAB-binding efficiency affects protein levels, we performed the Tandem Mass Tag (TMT)-based quantitative proteomics on the 2-week-old seedlings of Col and the *mut*, respectively (Fig. 5e). Genes with reduced protein synthesis rate per mRNA in the *mut* showed higher AtPAB-binding efficiencies (Fig. 5f, g; Additional file 1: Figure S10d) and higher fractions of exclusive-A tails (Fig. 5h, *P* = 0.005), again indicating the role of the “purity” of poly(A) tails and AtPAB binding in enhancing protein synthesis.

## Discussion

The poly(A) tail is a hallmark of the eukaryotic messenger RNA [1, 2]. It has been widely accepted that poly(A) tails are exclusively adenosines and regulate mRNA stability through the number of adenosines in the tail and probably the number of PABs bound to them. Recent studies reported that non-A nucleosides (especially guanosine) existed in the poly(A) tails of human cells [16–19]. In this study, we echoed in plants that guanosine was prevalent in poly(A) tails and furthermore demonstrated the role of G-content in regulating translational efficiency through AtPAB binding. Our study showcases the power of the next-generation genome-biology tools in understanding the basic principles underlying the central dogma.

We did not observe a significant effect of AtPAB-binding efficiency on mRNA stability, likely because the regulation of mRNA stability by its tail is a complex and dynamic process. On the one hand, PABs can protect mRNA from degradation by preventing deadenylase complex from poly(A) tails [39–41]. On the other hand, guanosines in poly(A) tails may slow down its trimming process mediated by deadenylase and consequently inhibit mRNA degradation [19]. Since guanosines negatively regulate PAB binding to the poly(A) tail, these two opposing effects could cancel out, leading to a net negotiable effect of PAB binding on the stability of mRNA (Fig. 4b).

We identified the role of AtPABs in translational enhancement. It could be related to the protein complex that PAB forms with translational initiation factors eIF4G and eIF4E. Such complex leads to a head-to-tail looping structure of mRNA [3, 42], which may promote translation through facilitating ribosome recycling [27, 29, 42]. More experiments are required to fully understand the mechanism.

The poly(A) tails are not static; rather, they are dynamically regulated during development and cell cycle [43–45]. For example, during the oocyte-to-embryo transformation in *Drosophila*, changes in the length of poly(A) tails were reported [43, 44]. Dramatic changes were also observed in the length of poly(A) tails during human somatic cell cycles [45]. It remains unclear whether the composition of the poly(A) tail, especially G%, is also variable in different cell states and contributes to translational regulation. It will be interesting to analyze the composition of poly(A) tail in various conditions, such as tissues, developmental stages, or environments. Such work will lead to a future direction for the ultimate understanding of poly(A)-tail-mediated regulation of gene expression [46–48].

## Conclusions

The *Arabidopsis thaliana pab* mutants exhibit various levels of phenotypic abnormality. Analysis of the RNA-binding landscape revealed a wide variation in AtPAB-binding efficiency among genes, which can be partly explained by the G% in the poly(A) tail. AtPAB binding enhances translation; genes with stronger binding to AtPABs exhibit more reduction in translational efficiency in the *atpab* mutant. These observations indicate that AtPABs can precisely enhance the translational efficiency of the genes with more uniform poly(A) tails.

## Methods

### Plasmid construction

The reporters for AtPABs were constructed in the background of a vector containing the GFP-HA tag (pCAMBIA1300 backbone). The promoters (2.0-kb regions upstream of the transcription start site) and genomic regions between the transcription start site and the site right before the stop codon were amplified with primers (cx8366 and cx8367 for *AtPAB2*; cx8370 and cx8371 for *AtPAB4*; cx8374 and cx8375 for *AtPAB8*) and were inserted before the GFP tag. The 3′-untranslated regions (UTRs) and terminator fragments (0.5 kb downstream to the transcription end site) were obtained by PCR amplification with primers (cx8368 and cx8369 for *AtPAB2*; cx8372 and cx8373 for *AtPAB4*; cx8376 and cx8377 for *AtPAB8*) and were inserted after the HA tag. Primers are listed in Additional file 2: Table S11.

### Plant materials

All *Arabidopsis thaliana* materials used in this study were in the Columbia (Col-0) background. The *atpab2-1* (SALK_026293), *atpab4-2* (SAIL_740_D08), and *atpab8-1* (SALK_022160) mutants were obtained from the Arabidopsis Biological Resources Center at Ohio State (https://abrc.osu.edu/). The primers used for genotyping are listed in Additional file 2: Table S11.

Seeds were sterilized in 75% ethanol for 1 min and treated with 10% bleach for 15 min. Seeds were sown on Murashige and Skoog (MS) media (0.43 g/L MS salts, 3 g/L sucrose, 0.8% agar, pH 5.8) and were treated at 4 °C for 3 days. Seeds were further grown at 23 °C under long-day conditions (cool-white fluorescent light on for 16 h and off for 8 h). Whole seedlings were harvested at 2 weeks for CLIP-seq, ribo-seq, poly(A)-seq, RNA stability assay, and mass spectrometry.

The transgenic plants *AtPAB2-GFP-HA atpab2 atpab8*, *AtPAB8-GFP-HA atpab2 atpab8*, and *AtPAB4-GFP-HA atpab2 atpab4* were generated by transforming *AtPAB* reporters into the double mutants with the floral dipping method [49]. MS plates containing 25 μg/mL hygromycin were used for the selection of transgenic plants. For each *AtPAB*, multiple single-copy insertion lines were obtained (Additional file 1: Figure S3c).

Plants were planted on soil mix (2/3 vermiculite and 1/3 nutrient soil) under long-day conditions for phenotyping. The total leaf number (including both rosette and cauline leaves) was used for the quantification of flowering time.

### Microscopy

To identify phenotypic abnormality in embryos, seeds were cleared in Herr’s solution (lactic acid:chloral hydrate:phenol:clove oil:xylene, 2:2:2:2:1, w/w) for 5–30 min until the embryonic morphology was clear under the microscope (Olympus BX51) in the differential interference contrast mode.

### EMSA

We followed the protocol of EMSA/Gel-Shift Kit (Beyotime, GS002) with modifications. The oligo-RNAs were synthesized chemically by Genscript (Nanjing, China). Oligo(A)-RNA was labeled with γ-^32^P-ATP by T4 polynucleotide kinase (T4 PNK, NEB, M0201) in the reaction mix (1000 Ci γ-^32^P-ATP, 3.5 pmol oligo(A)-RNA, 10 U T4 PNK, 1 × T4 PNK buffer) at 37 °C for 10 min. For each EMSA reaction, 1% labeled oligo(A)-RNA probe (~ 0.035 pmol) was incubated with ~ 100 ng AtPAB protein at 25 °C for 10 min in a 10-μL reaction system. The reaction mix contains 4% glycerol, 1 mM MgCl_2_, 0.5 mM EDTA, 0.5 mM DTT, 50 mM NaCl, 10 mM Tris-HCl (pH 7.5), and 0.05 mg/mL poly (dI-dC)•poly (dI-dC). The ^32^P-labeled oligo(A)-RNA was added together with the unlabeled competing oligo-RNA. The reaction mix was separated by native polyacrylamide gel (1 × Tris-glycine buffer, 5% glycerol, 6% polyacrylamide) electrophoresis (PAGE) in the 2 × Tris-glycine buffer at 80 V for 1 h. 1 × Tris-glycine buffer contains 25 mM Tris (pH 8.5), 190 mM glycine, and 1 mM EDTA. The gel was wrapped in plastic film and exposed to a storage phosphor screen (GE Healthcare) overnight, and signals were read with a Typhoon TRIO scanner (GE Healthcare).

### CLIP-seq and RIP-RT-PCR

CLIP-seq was performed as described previously [33]. In brief, to cross-link RNA and protein, 2-week-old seedlings were soaked in ice-cold PBS buffer and were UV-treated twice, each with the irradiation intensity at 600 mJ/cm^2^ (Hoefer UVC 500 Ultraviolet Crosslinker, GE). The RNA-AtPAB-GFP-HA complexes were enriched from the lysate by immunoprecipitation using anti-HA antibody (Sigma, H6908) and were partially digested by micrococcal nuclease (3 × 10^−5^ U/μL, Fermentas, EN0181). The digested RNA was ligated to the 3′-RNA adaptor (P-UGGAAUUCUCGGGUGCCAAGGUidT) and was labeled by ^32^P at the 5′-end. The radioactive RNA-protein complexes (RNP) were separated by SDS-PAGE and were further transferred to nitrocellulose membrane. Membrane pieces corresponding to the RNP-containing radioactive regions (Additional file 1: Figure S5b, c) were selected to recover RNA fragments. RNPs were treated with Proteinase K, and the purified RNA was ligated to the 5′-RNA adaptor (GUUCAGAGUUCUACAGUCCGACGAUCNNNN).

The immunoprecipitation in RIP-RT-PCR was same as that in CLIP-seq, but the RNA-protein complexes were not digested by micrococcal nuclease. qPCR was performed on four genes to quantify the mRNA abundance in a sample. The AtPAB2-binding efficiency of a gene was estimated from its mRNA abundance in the AtPAB2-IP product, normalized by its abundance in the total mRNA. Primers for RIP-RT-PCR are listed in Additional file 2: Table S11.

### Poly(A)-seq

Total RNA was extracted from the 2-week-old seedlings of Col. Five micrograms of total RNA was treated with RQ1 DNase (Promega, M6101) and was partially digested by RNase T1 (Thermo, EN0541), an endoribonuclease that specifically digests single-stranded RNA at the 3′-side of the G-residue. Poly(A)-containing RNA was purified with oligo (dT)-conjugated magnetic beads (Invitrogen, 61005) and ligated to the 3′-RNA adaptor (TGGAATTCTCGGGTGCCAAGG). The cDNA was generated using the ScriptSeq v2 RNA-Seq Library Preparation Kit (Illumina, SSV21124). PCR products of 200–500 bp were purified and were applied to HiSeq X ten system for 150-nt paired-end sequencing.

### mRNA stability assay

Two-week-old seedlings (0.1 g) were transferred to 2 mL incubation buffer (1 mM PIPES, pH 6.25, 1 mM sodium citrate, 1 mM KCl, 15 mM sucrose) in a 24-well plate. Cordycepin was added to a final concentration of 0.5 mM. The plate was rotated for 75 rpm at 23 °C. The seedlings were harvested at 0, 0.5, 1, 2, 4, and 8 h and quickly frozen in liquid nitrogen. Total RNA was isolated with TRIzol (Invitrogen, 15596026). The mRNA-seq libraries were constructed using the strand-specific protocol for Illumina sequencing and were sequenced with the HiSeq X ten system in the paired-end 150-nt mode.

### Ribo-seq

The protocol was modified from a previous study [38]. In brief, ribosomes were extracted from 5-g 2-week-old seedlings with the polysome extraction buffer (PEB), which contains 200 mM Tris-HCl (pH 9.0), 200 mM KCl, 25 mM EGTA, 35 mM MgCl_2_, 1% (w/v) polyoxyethylene(23) lauryl ether (Brij-35), 1% (v/v) Triton X-100, 1% (v/v) octylphenyl-polyethylene glycol (Igepal CA 630), 1% (v/v) polyoxyethylene sorbitan monolaurate 20 (Tween 20), 1% (v/v) polyoxyethylene(10) tridecyl ether, 5 mM DTT, 1 mM PMSF, 50 μg/mL cycloheximide, and 50 μg/mL chloramphenicol. The extracted ribosomes were pelleted through a 30-mL sucrose cushion containing 400 mM Tris-HCl (pH 9.0), 200 mM KCl, 5 mM EGTA, 30 mM MgCl_2,_ 1.75 M sucrose, 5 mM DTT, 50 μg/mL cycloheximide, and 50 μg/mL chloramphenicol, by ultracentrifugation at 4 °C overnight (33,500 rpm, Beckman, 70Ti rotor). Ribosome-protected mRNA fragments were generated by RNase I (Ambion, AM2294) digestion and were applied to small RNA library construction for Illumina sequencing (Gnomegen, k02420).

### Polysome profiling

The ribosomes were extracted as described in the ribo-seq experiments and were separated through a 5–50% sucrose gradient by ultracentrifugation at 4 °C and 35,300 rpm (SW41 rotor, Beckman) for 3 h. The profiling signals were detected using a piston gradient fractionator (Biocomp, B152-002) at 254-nm UV absorbance.

### Mass spectrometry

Two-week-old *Arabidopsis* seedlings (~ 2 g each) were ground in the liquid nitrogen, and the total protein was extracted with trichloroacetate. One hundred fifty micrograms of protein of each sample was run into the SDS-PAGE as gel plug and digested with trypsin at 37 °C overnight. Seventy-five micrograms of protein of each sample was labeled with TMT 6plex reagent (Thermo) and combined after labeling and dried.

Samples were desalted with a SPEC C18 column and solubilized in 200 μL buffer A (20 mM ammonium formate, pH 10) and separated on an Xbridge column (Waters; C18; 3.5 μm, 2.1 × 150 mm) using the Agilent HP1100. Fractions were collected at 1-min intervals and dried under vacuum. The LC-MS/MS/MS (liquid chromatography-tandem mass spectrometry) analysis was performed using a Dionex rapid-separation liquid chromatography system interfaced with an Orbitrap Fusion™ Lumos™ mass spectrometer (Thermo Scientific).

### Bioinformatics analyses

#### Analyses of mRNA-seq data

The 3-nt random sequence at the 5′-end of the clean reads was removed. The trimmed clean reads were aligned to the *Arabidopsis* genome (TAIR10) using TopHat2 (2.0.11) [50] with no more than five mismatches. The expression level of each gene was estimated from the uniquely mapped fragments using HTSeq (0.6.0) [51] and was normalized to the unit of fragments per kilobase of transcript per million mapped fragments (FPKM). Two biological replicates were highly correlated (*r* > 0.99, *P* < 10^−100^, Pearson’s correlation, Additional file 2: Table S8).

#### Analyses of CLIP-seq data

Single-end clean reads with identical sequences (including the 4-nt random sequence in the 5′-RNA adaptor) were defined as PCR duplicates and were reduced to 1 read. The 4-nt random sequence at the 5′-end and the 3′-adaptor sequence of Illumina were removed using the FASTX toolkit (0.0.14) (http://hannonlab.cshl.edu/fastx_toolkit). The 3′-end A-tracts in each read were removed, and the trimmed reads of less than 18 nt in length were discarded. The remaining reads were mapped to the *Arabidopsis* genome (TAIR10) using NovoAlign (3.07.01) (Novocraft, http://www.novocraft.com/), allowing up to two mismatches. Uniquely mapped reads were used to identify AtPAB binding clusters using the Pyicos toolkit (2.0.7) [34]. The AtPAB-binding efficiency of a gene was calculated as the ratio between the number of reads (per million mapped reads) in the CLIP-seq and that of fragments (FPKM) in the mRNA-seq of the wild-type.

#### Analyses of poly(A)-seq data

To avoid the problem of polymerase slip on a homopolymeric tract, we only used high-quality 5′-sequencing reads with recognizable 3′-adaptor sequences of Illumina sequencing for analyses. The 3-nt random sequence at the 5′-end and 3′-adaptor sequence of Illumina were removed. The poly(A) tail was defined as a stretch of A’s at the 3′-end of a read, allowing up to 5 interspersed G bases. We also required at least 5 A’s at the 5′-end of a poly(A) tail. The poly(A)-tail sequences were removed, and trimmed reads of at least 18 nt in length were aligned to the *Arabidopsis* genome (TAIR10) using Bowtie2 (2.3.4.1) [52], allowing up to two mismatches. Consequently, the sequences of a poly(A) tail and the gene it belongs to were identified. We confirmed manually that the majority of the G’s (500 out of the 500 manually checked G’s) in the identified poly(A) tails could not be explained by the G’s in the 3′-UTR. For each read, G% in the poly(A) tail was estimated as the number of G’s divided by the length of the poly(A) tail. The G% of a gene was calculated as the total number of G’s divided by the total length of poly(A) tails. The poly(A)-tail length of each gene was estimated with the median length of all its poly(A) tails.

#### Analyses of mRNA stability assay data

Clean mRNA-seq reads were aligned to the *Arabidopsis* genome (TAIR10) using TopHat2 (2.0.11) [50], allowing up to two mismatches. The expression level of each gene was estimated using Cufflinks (2.2.1) [53], in the unit of fragments per kilobase of transcript per million mapped fragments (FPKM). The mRNA degradation rate of each gene was defined as the slope of the regression line in the linear model ln (mRNA_0_/mRNA_*t*_) ~ *t*. Three biological replicates were highly correlated (*r* > 0.9, *P* < 10^−100^, Pearson’s correlation, Additional file 2: Table S8), and therefore, the average mRNA degradation rate was used for further analyses.

#### Analyses of ribo-seq data

Reads with identical sequences (including the 4-nt random sequence at the 5′-end) were likely PCR duplicates and were reduced to 1 read. The 3′-end adaptors and 5′-end 4-nt barcodes were removed. Reads were mapped to the *Arabidopsis* genome (TAIR10) using TopHat2 (2.0.11) [50], allowing no more than two mismatches. The number of reads per kilobase of the coding sequence per million mapped reads (RPKM) was estimated for each gene. Genes with > 0.5 RPKM in ribo-seq and > 1 FPKM in mRNA-seq were kept for the calculation of translational efficiency. The translational efficiency of a gene was calculated as the ratio between the average number of reads (RPKM) in the Ribo-seq and that of fragments (FPKM) in the mRNA-seq. The translational efficiency was highly reproducible among replicates (Additional file 2: Table S8).

#### Analysis of mass spectrometry data

All data were analyzed with MaxQuant (version 1.6.2.6) with Andromeda search engine [54]. The type of LC-MS run was set to reporter ion MS3 with 6plex TMT as isobaric labels. LC-MS data were searched against TAIR10 database with the addition of potential contaminants.

## Additional files

Additional file 1: **Figure S1.** *AtPAB2*, *AtPAB4*, and *AtPAB8* are constitutively expressed in Arabidopsis. **Figure S2.** The mutants of *AtPABs* exhibited severe phenotypes. **Figure S3.** The expression of *AtPAB-GFP-HA* rescued the developmental defects of *atpab* double mutants. **Figure S4.** AtPAB-GFP-HA reporters were ubiquitously and constitutively expressed in the cytoplasm during seed development. **Figure S5.** Identification of AtPAB-binding targets. **Figure S6.** The AtPAB-binding genes detected in CLIP-seq were validated by RIP-RT-PCR. **Figure S7.** AtPABs bind the poly(A) tails of mRNAs with different efficiencies. **Figure S8.** G% in the poly(A) tail is a determinant of the AtPAB-binding efficiency. **Figure S9.** The major consequence of AtPAB-binding is translational enhancement. **Figure S10.** AtPAB binding enhances translation efficiency. (PDF 1987 kb)

Additional file 2: **Table S1.** Progeny segregation from the self-cross of the *mut* (*atpab2*^*+/−*^ *atpab4 atpab8* plants). **Table S2.** Number of normal and aborted seeds in the mature *mut* siliques. **Table S3.** Number of embryos at different developmental stages in each Col silique. **Table S4.** Number of embryos at different developmental stages in each *mut* silique. **Table S5.** Summary of the CLIP-seq reads. **Table S6.** Summary of the poly(A)-seq reads. **Table S7.** Summary of reads of the mRNA stability assay. **Table S8.** Correlations between biological replicates. **Table S9.** Summary of the ribo-seq reads. **Table S10.** Summary of the mRNA-seq reads. **Table S11.** List of primer sequences. (PDF 474 kb)

## Data Availability

The *Arabidopsis* genome (TAIR10) was retrieved from https://www.arabidopsis.org/ [55]. The mRNA level of *AtPABs* in multiple tissues was calculated with the data obtained from Expression Altas (https://www.ebi.ac.uk/gxa/home) [56]. Paralogous gene pairs in *Arabidopsis* were retrieved from Ensembl plants with BioMarts [57]. High-throughput sequencing data of CLIP-seq, ribo-seq, mRNA-seq, poly(A)-seq, and RNA-stability-seq have been deposited to the Gene Expression Omnibus database under accession number GSE110342 [58]. The mass spectrometry proteomics data have been deposited to the ProteomeXchange Consortium via the PRIDE [59, 60] partner repository with the dataset identifier PXD014071 [61]. Codes to analyze the data and to generate figures are available at GitHub [62] and Zenodo [63].
