# Supplementary material for: Impact of poly(A)-tail G-content on Arabidopsis PAB binding and their role in enhancing translational efficiency
Source: Genome Biol. 2019 Sep 3;20:189. doi: 10.1186/s13059-019-1799-8 (PMC6724284; doi:10.1186/s13059-019-1799-8)
Supplement: Supplementary file 1 — Figure S1. AtPAB2, AtPAB4, and AtPAB8 are constitutively expressed in Arabidopsis. Figure S2. The mutants of AtPABs exhibited severe phenotypes. Figure S3. The expression of AtPAB-GFP-HA rescued the developmental defects of atpab double mutants. Figure S4. AtPAB-GFP-HA reporters were ubiquitously and constitutively expressed in the cytoplasm during seed development. Figure S5. Identification of AtPAB-binding targets. Figure S6. The AtPAB-binding genes detected in CLIP-seq were validated by RIP-RT-PCR. Figure S7. AtPABs bind the poly(A) tails of mRNAs with different efficiencies. Figure S8. G% in the poly(A) tail is a determinant of the AtPAB-binding efficiency. Figure S9. The major consequence of AtPAB-binding is translational enhancement. Figure S10. AtPAB binding enhances translation efficiency. (PDF 1987 kb) [file 13059_2019_1799_MOESM1_ESM.pdf]

SUPPLEMENTARY FIGURES

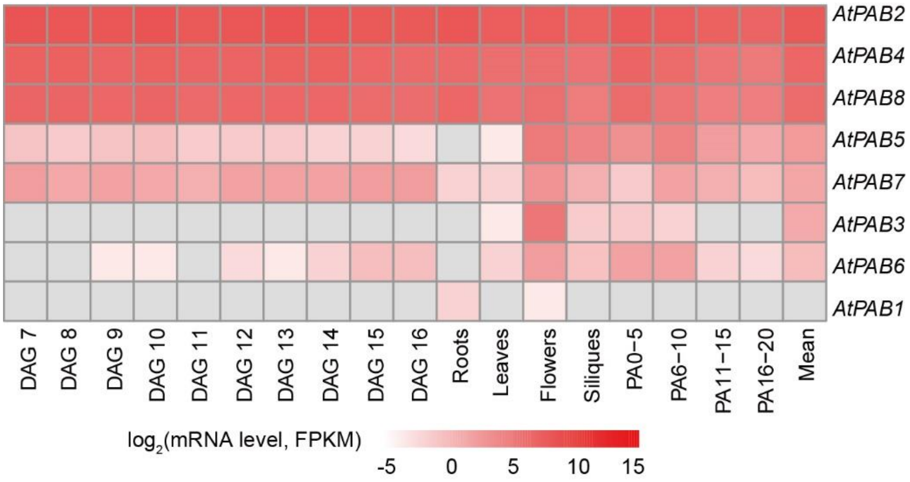

Figure S1. ***AtPAB2*, *AtPAB4*, and *AtPAB8* are constitutively expressed in *Arabidopsis*.**
Heatmap shows the expression level of eight *AtPAB* family members in different tissues and developmental stages (DAG: days after germination; PA: post-anthesis). The mRNA level of *AtPABs* was calculated with data obtained from Expression Altas (https://www.ebi.ac.uk/gxa/home) under the number E-MTAB-4202 (during *Arabidopsis* meristem development from day 7 to 16 after germination), E-GEOD-38612 (roots, leaves, flowers, and siliques), and E-GEOD-55866 (siliques at four developmental stages).

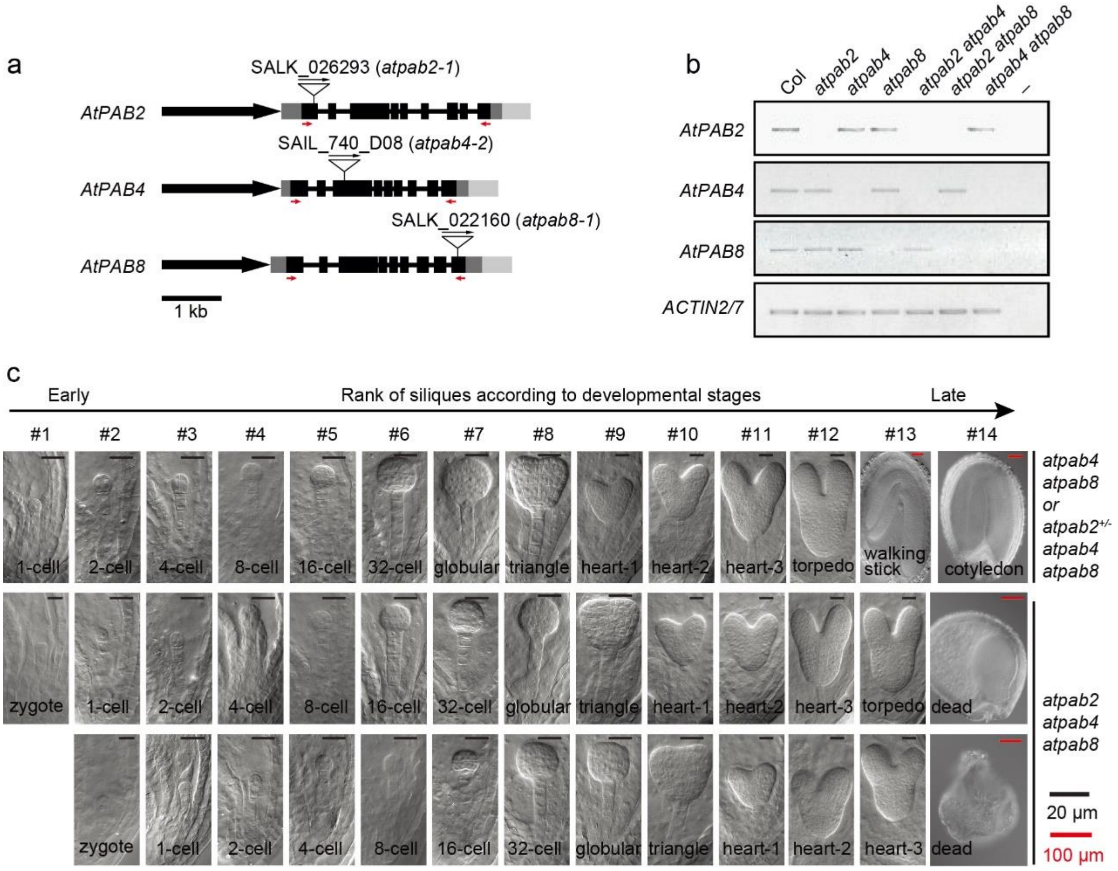

Figure S2. The mutants of *AtPABs* exhibited severe phenotypes.

a Gene models of *AtPAB2*, *AtPAB4*, and *AtPAB8* are shown, including promoters (long black arrows), untranslated regions (dark grey boxes), exons (black boxes), introns (black lines), and terminators (light grey boxes). T-DNA insertion sites and directions are marked by triangles and arrows, and the ABRC (*Arabidopsis* Biological Resource Center) accession numbers are labeled.

b RT-PCR analysis of the expression of *AtPAB2*, *AtPAB4*, and *AtPAB8* in the *atpab* mutants. *ACTIN2/7* was used as an internal control. The minus sign (–) represents the PCR negative control. Primers used for RT-PCR are marked in **a** by red arrows.

c Asynchronous development of embryos was observed in the *mut* siliques. Embryos in each column were obtained from the same silique of the *mut*.

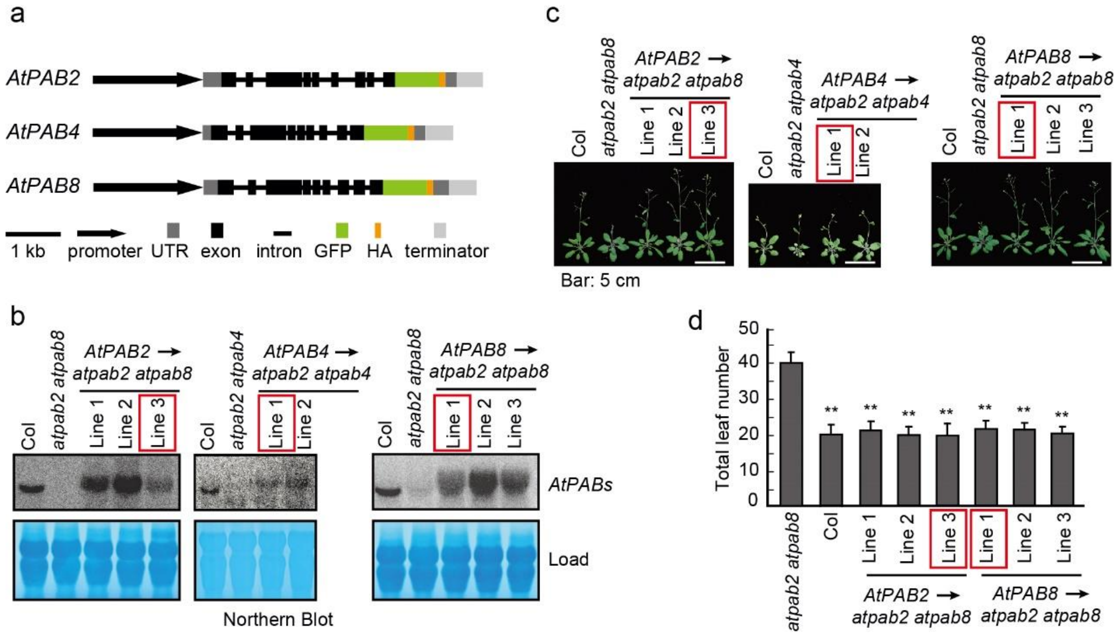

Figure S3. The expression of *AtPAB-GFP-HA* rescued the developmental defects of *atpab* double mutants.

a Models show the structures of the *AtPAB* reporters.

b The expression level of *AtPABs* was validated by Northern blot in their transgenic lines. The methylthionine chloride-stained total RNAs were used as loading controls. Transgenic lines used for CLIP-seq were labeled by red rectangles.

c The developmental defects of *atpab* double mutants were rescued by the expression of *AtPABs* in the transgenic lines.

d The late-flowering phenotype of *atpab2 atpab8* was rescued by the expression of *AtPAB2* or *AtPAB8*. Plants were cultivated under long-day conditions. The total leaf number was counted for each plant. The average of more than 15 plants of each line is shown. Error bars represent standard errors. *P*-value was given by the Student's *t*-test, and the double asterisks indicate $P < 0.01$.

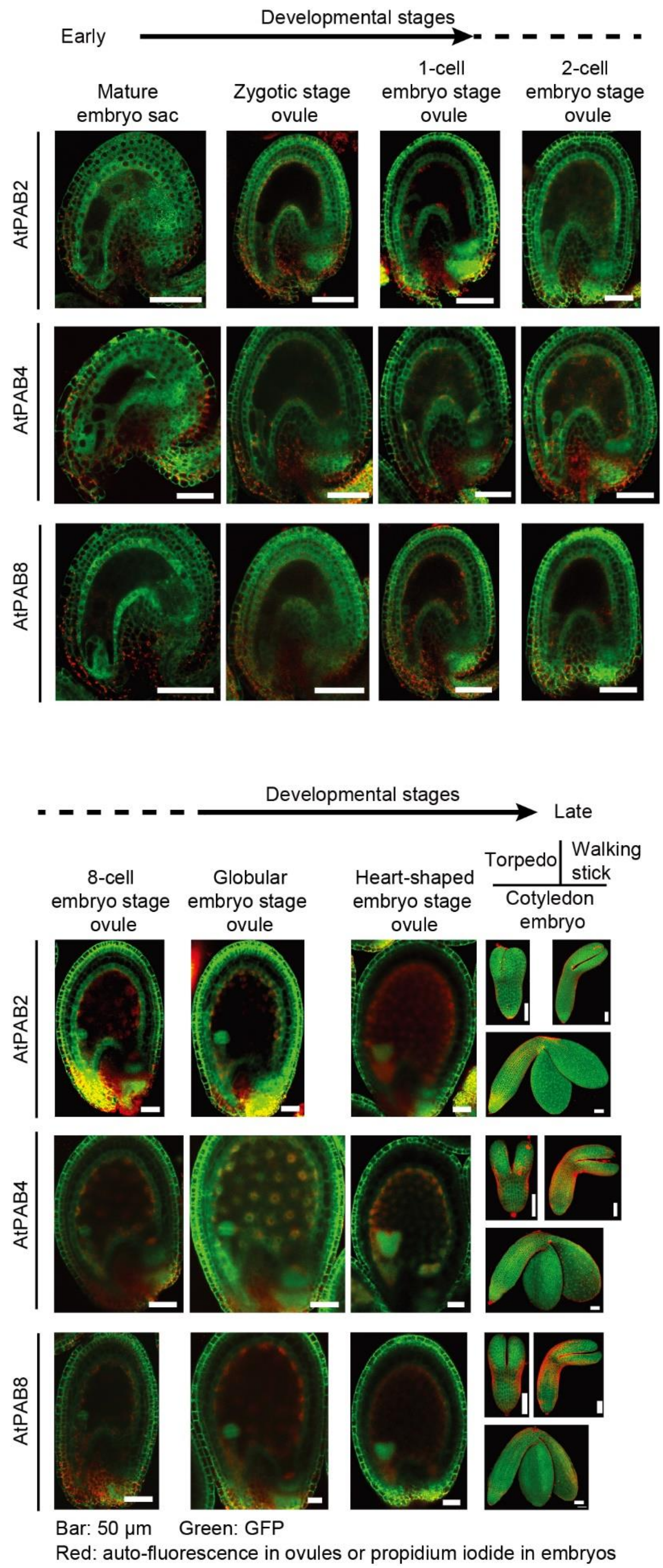

Figure S4. AtPAB-GFP-HA reporters were ubiquitously and constitutively expressed in the cytoplasm during seed development.

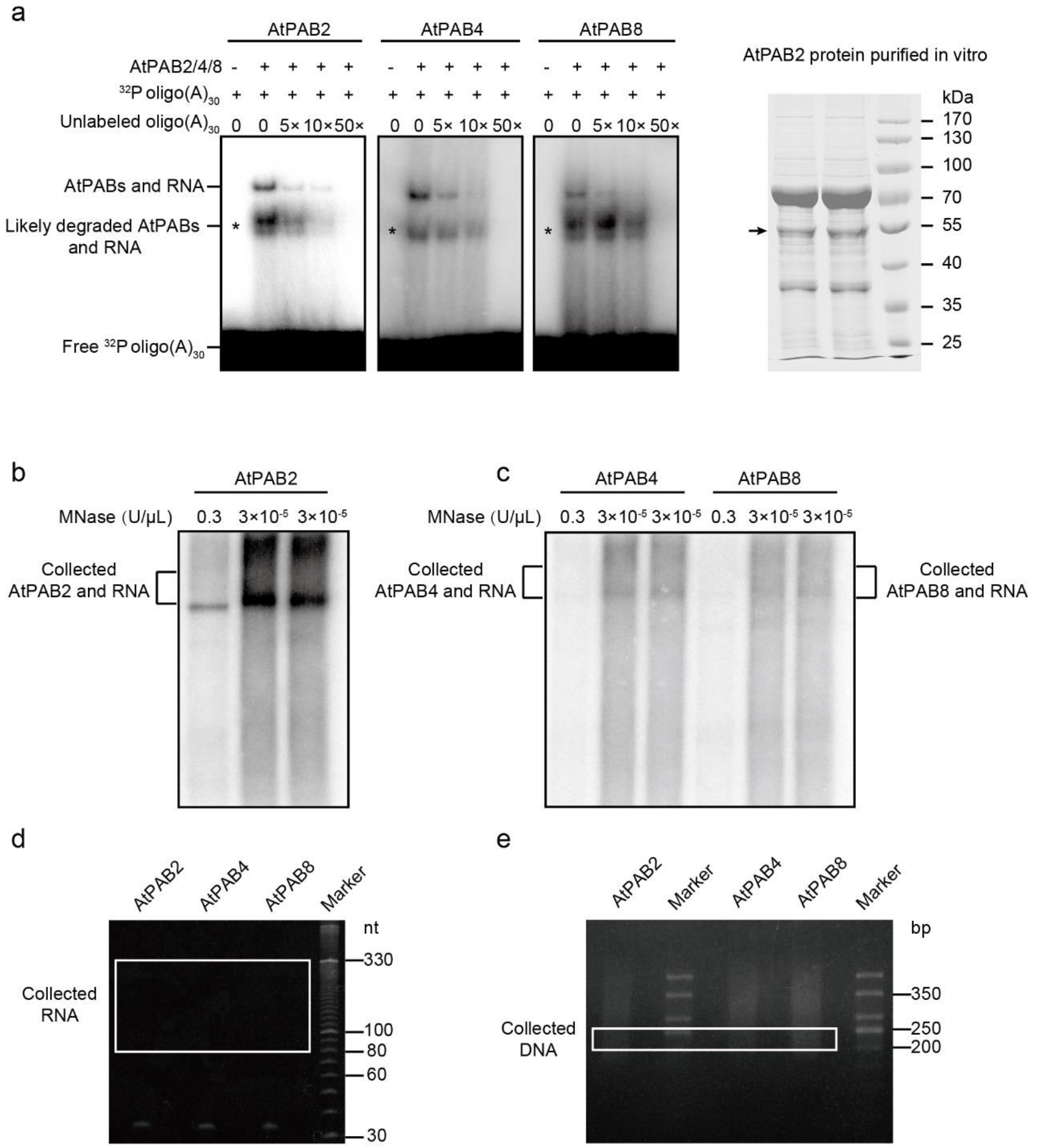

Figure S5. Identification of AtPAB-binding targets.
a AtPAB2, AtPAB4, and AtPAB8 bind oligo(A)$_{30}$ *in vitro* (**left panel**). "*n*×" means the amount of unlabeled oligo(A)$_{30}$ added was *n* times as much as that of ^{32}P oligo(A)$_{30}$. We inferred the asterisk marked bands as degraded AtPABs-RNA, because the AtPAB protein used here contains a protein band with the molecular weight (marked by an arrow) similar to its RNA-binding domain (**right panel**), which is sufficient for poly(A) binding. Another possibility is that the upper band contains AtPAB dimer and the lower contains AtPAB monomer. Considering one PAB generally protects ~25 As (Sachs et al., 1987), the degradation hypothesis is preferred. In either scenario, the conclusion that AtPABs bind to poly(A) hold.
b–e Construction of CLIP-seq libraries. PAGE (polyacrylamide gel electrophoresis) of the MNase treated immunoprecipitation (IP) products of AtPAB2 (**b**), AtPAB4, and AtPAB8 (**c**). The IP products were completely digested with MNase (0.3 U/μL) or partially digested (3×10^{-5} U/μL). The products of partial digestion in the marked

regions were collected for the library construction.

d PAGE separation after the 5′-RNA adapter ligation. RNA fragments of 80–300 nt (inside the white rectangle) were recovered from the gel. The amount of RNA here was too low to be observed.

e Agarose gel separation of the final PCR products. DNA library with 200–250 bp in length (inside the white rectangle) was selected for sequencing.

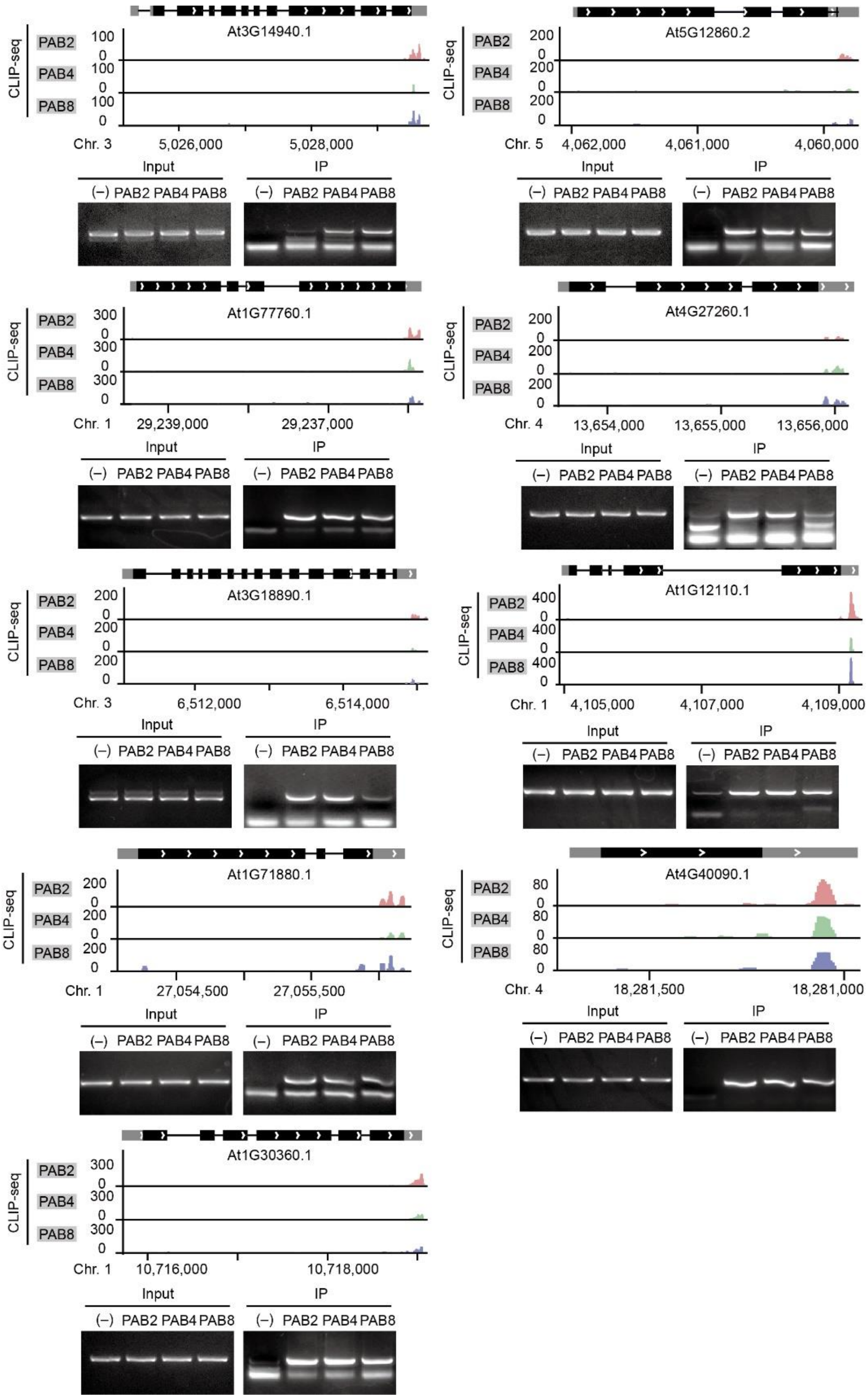

Figure S6. The AtPAB-binding genes detected in CLIP-seq were validated by RIP-RT-PCR.

The distribution of the AtPAB-CLIP reads is shown by the wiggle plots. The gene model shows the untranslated regions (gray boxes), coding sequences (black boxes), and introns (lines). The input and IP panels show the mRNA level of a gene in total RNA and RIP experiments (using anti-HA antibody), respectively. The minus sign (–) indicates the negative control (the wild-type Col) in which the GFP-HA tagged AtPAB is absent.

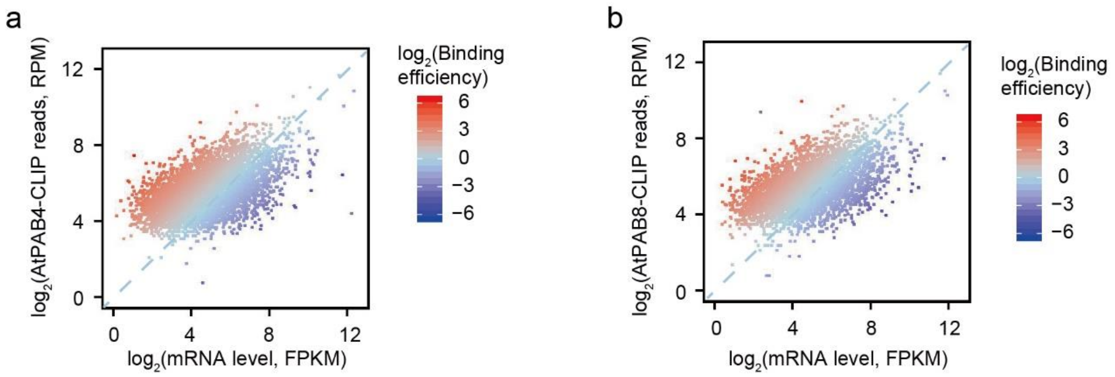

Figure S7. AtPABs bind the poly(A) tails of mRNAs with different efficiencies.
a–b The AtPAB4/8-binding efficiency significantly varies among genes. Each dot represents a target gene of AtPAB4/8.

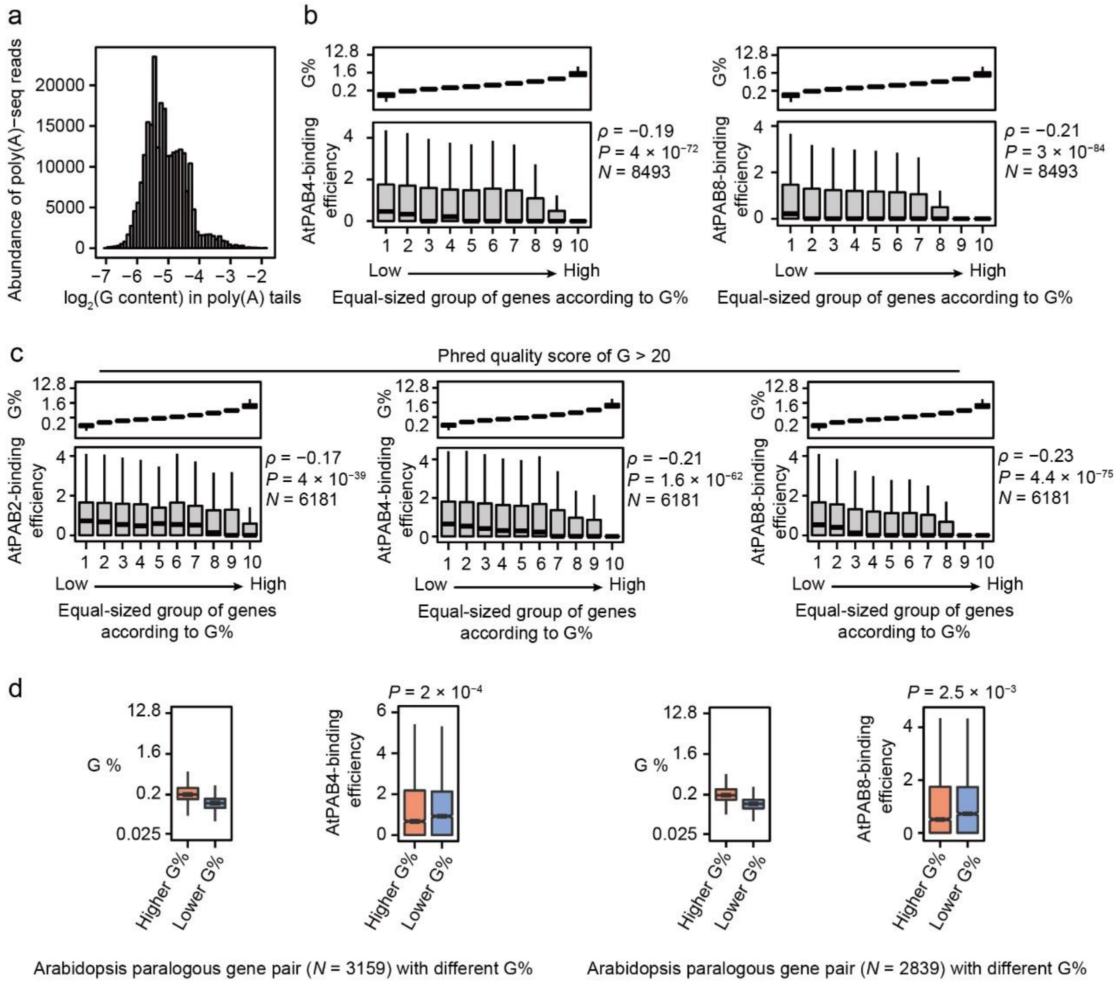

Figure S8. G% in the poly(A) tail is a determinant of the AtPAB-binding efficiency.

a Histogram shows the distribution of guanosine content in the poly(A) tails containing at least one guanosine.

b The guanosine content (G%) in a gene was negatively correlated with the AtPAB4/8-binding efficiency of the gene. Genes were divided into ten equal-size groups according to the average G% of the poly(A) tails of a gene. *P*-values were given by the Spearman's correlation.

c Similar to **b**, but removing reads with the quality score of at least one G <20.

d Paralogous genes with decreased G% in the poly(A) tails exhibit higher AtPAB4/8-binding efficiencies than their within-species paralogs with increased G% in the poly(A) tails. *P*-values were given by the paired Mann-Whitney *U* test.

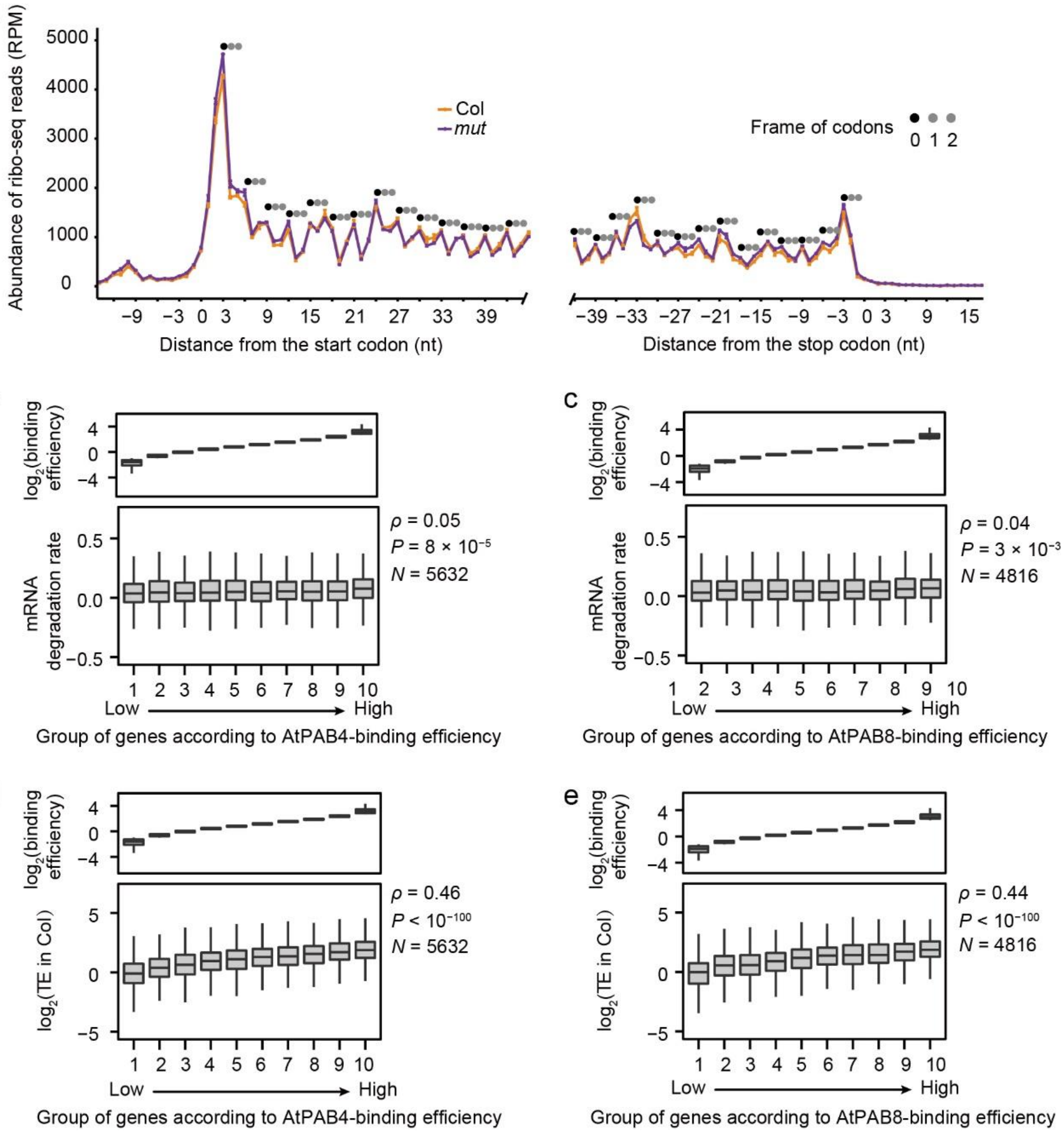

Figure S9. The major consequence of AtPAB-binding is translational enhancement.

a The number of ribo-seq reads falling around the start and stop codons of protein-coding genes in the genome. The position of the 17th nucleotide in a read was used, which represents the 5′-end nucleotide in the A site of a ribosome. The frame of codons was marked by dots with distinct colors. Error bars represent standard errors of the mean.

b–c The AtPAB4/8-binding efficiency was poorly correlated with the mRNA degradation rate. Genes were divided into ten equal-size groups according to the AtPAB4/8-binding efficiency. *P*-values were given by the Spearman's correlation.

d–e The AtPAB4/8-binding efficiency was positively correlated with translational efficiency (TE). Genes were divided into ten equal-size groups according to the AtPAB4/8-binding efficiency. *P*-values were given by the Spearman's correlation.

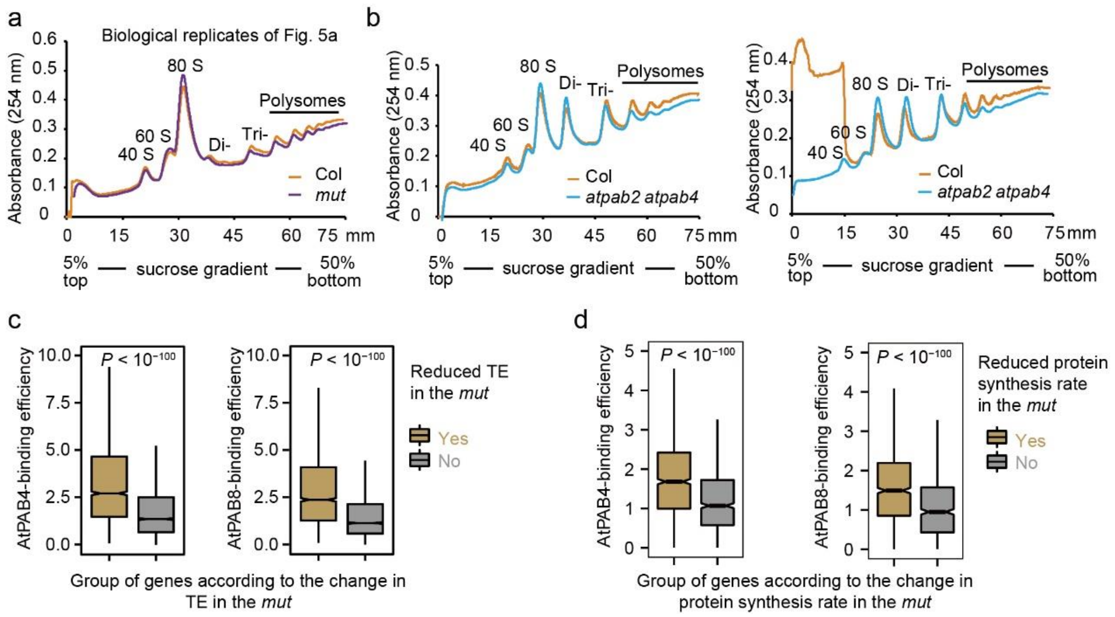

Figure S10. AtPAB binding enhances translation efficiency.

a Polysome profiling shows the global down-regulation of TE in the two-week-old seedlings of the *mut*. This is a biological replicate of **Fig. 5a**.

b Polysome profiling shows the global down-regulation of TE in the six-week-old plants of *atpab2 atpab4*.

c Genes with a significant reduction of TE in the *mut* (**Fig. 5b**) exhibited significantly higher AtPAB4/8-binding efficiencies. *P*-values were given by the Mann-Whitney *U* tests.

d Genes with a significant reduction of protein synthesis rate in the *mut* (**Fig. 5f**) exhibited significantly higher AtPAB4/8-binding efficiencies. *P*-values were given by the Mann-Whitney *U* tests.
