# Supplementary material for: Impact of poly(A)-tail G-content on Arabidopsis PAB binding and their role in enhancing translational efficiency
Source: Genome Biol. 2019 Sep 3;20:189. doi: 10.1186/s13059-019-1799-8 (PMC6724284; doi:10.1186/s13059-019-1799-8)
Supplement: Supplementary file 2 — Table S1. Progeny segregation from the self-cross of the mut (atpab2+/− atpab4 atpab8 plants). Table S2. Number of normal and aborted seeds in the mature mut siliques. Table S3. Number of embryos at different developmental stages in each Col silique. Table S4. Number of embryos at different developmental stages in each mut silique. Table S5. Summary of the CLIP-seq reads. Table S6. Summary of the poly(A)-seq reads. Table S7. Summary of reads of the mRNA stability assay. Table S8. Correlations between biological replicates. Table S9. Summary of the ribo-seq reads. Table S10. Summary of the mRNA-seq reads. Table S11. List of primer sequences. (PDF 474 kb) [file 13059_2019_1799_MOESM2_ESM.pdf]

SUPPLEMENTARY TABLES

Table S1. Progeny segregation from the self-cross of the *mut* (*atpab2*$^{+/-}$ *atpab4 atpab8* plants).

Parental genotype	Progeny genotype					
	AtPAB2 +/+	*AtPAB2* +/-	*AtPAB2* -/-	Segregation ratio between *AtPAB2*$^{+/+}$ and *AtPAB2*$^{+/-}$	Expected ratio	*P* value [a]
atpab2$^{+/-}$ *atpab4 atpab8*	43	77	0	1:1.79	1:2	0.56

[a] *P* value was given by the binomial exact test.

Table S2. Number of normal and aborted seeds in the mature *mut* siliques.

Number of normal seeds	Number of aborted seeds	Aborted/total	Segregation ratio	Expected ratio	*P* value [a]
2552	885	25.73%	2.88:1	3:1	0.33

[a] *P* value was given by the binomial exact test.

Table S3. Number of embryos at different developmental stages in each Col silique.

Developmental stage	Silique													
	1	2	3	4	5	6	7	8	9	10	11	12	13	14
**Zygote**														
**1 cell**				1										
**2 cell**	11													
**4 cell**	20													
**8 cell**	14	3		1										
**16 cell**		30	1											
**32 cell**		11	3	1										
**Globular**		1	38	9										
**Triangle**				37	1									
**Heart**					39									
**Torpedo**						49	54							
**Walking stick**								55						
**Early cotyledon**									54	56	52			
**Cotyledon**												56	55	55
**Total**	45	45	42	49	40	49	54	55	54	56	52	56	55	55

The top row labels the siliques in the order from the youngest (the smallest rank) to the oldest (the largest rank). The first column shows different embryo developmental stages. Embryos at different developmental stages were counted in individual siliques. The total number of embryos in each silique is listed in the bottom line.

Table S4. Number of embryos at different developmental stages in each *mut* silique.

Developmental stage	Silique													
	1	2	3	4	5	6	7	8	9	10	11	13	15	21
**Zygote**	7													
**1 cell**	21	3												
**2 cell**	11	7												
**4 cell**	1	18		1										
**8 cell**		12	4	1										
**16 cell**		2	17	4	3									
**32 cell**			17	11	6									
**Globular**			6	12	12	1						1		
**Triangle**				7	19	3	6		1					
**Heart**					9	18	30	30	15	7	9	4	9	
**Torpedo**					1	20		12	27	12	4			9
**Walking stick**										21	23			
**Cotyledon**												32	31	30
**Total**	40	42	44	36	50	44	36	42	43	40	36	37	40	39

Similar to Table S3.

Table S5. Summary of the CLIP-seq reads.

Library	Total reads	Non-poly(A) reads [a]	Filtered reads [b]	Uniquely mapped reads [c]	Reads mapped to 3′-UTRs	
					Clusters [d]	Genes
**AtPAB2**	16920678	3124160	1873818	1401428	8857	8322
**AtPAB4**	21583503	2150081	1867296	1180564	7311	6292
**AtPAB8**	16985860	2532515	1976272	970455	6300	5356

[a] Number of reads after removing reads with exclusive A's and the PCR duplicates. [b] Number of reads of at least 18 nt in length after trimming the 3′-adaptor sequence, the 4-nt random sequence at the 5′-end, and the 3′-end A-tract. [c] Number of reads uniquely mapped to the *Arabidopsis* genome (TAIR10). [d] Consecutive sites with an mFDR <0.01 and ≥10 reads were identified as significant binding clusters using the Pyicos toolkit.

Table S6. Summary of the poly(A)-seq reads.

Library	Total reads	Clean reads [a]	Reads with recognizable 3′-adaptor [b]	Uniquely mapped reads [c]
**Col**	34601317	34368235	11736593	2936540

[a] Number of reads with an average Phred quality score greater than 30. [b] Number of reads with recognizable 3′-adaptor sequences of the Illumina library. [c] Number of reads uniquely mapped to the *Arabidopsis* genome (TAIR10).

Table S7. Summary of reads of the mRNA stability assay.

Libraries	Total reads	Clean reads [a]	Mapped reads [b]	Uniquely mapped reads [c]
**Col 0h-rep1**	13922489	13837980	13057548	12657670
**Col 0h-rep2**	14737567	14609603	13696143	13307591
**Col 0h-rep3**	13499858	13363347	12511950	12132247
**Col 0.5h-rep1**	16567796	16414157	15551435	15154066
**Col 0.5h-rep2**	15759651	15666655	14717065	14340717
**Col 0.5h-rep3**	14087549	14004856	13146061	12784576
**Col 1h-rep1**	15537706	15432085	14593535	14076143
**Col 1h-rep2**	13358211	13281288	12466055	12154395
**Col 1h-rep3**	15956029	15833329	14808032	14370399
**Col 2h-rep1**	15971797	15885070	15008792	14081304
**Col 2h-rep2**	15210183	15124115	14173336	13741551
**Col 2h-rep3**	13429959	13320382	12496719	12196549
**Col 4h -rep1**	14705678	14648685	13862157	13139559
**Col 4h-rep2**	17162086	17040913	16065451	15535271
**Col 4h-rep3**	15006973	14886989	13916250	13458243
**Col 8h-rep1**	14663284	14596099	13738772	12800723
**Col 8h-rep2**	12923459	12851281	11969133	11435980
**Col 8h-rep3**	13359572	13287502	12356746	11701766

[a] Number of reads with an average Phred quality score greater than 20. [b] Number of reads mapped to the *Arabidopsis* genome (TAIR10). [c] Number of uniquely mapped reads.

Table S8. Correlation between biological replicates.

Samples	Pearson's correlation coefficient
**mRNA stability assay**	
Col 0h rep1 vs. Col 0h rep2	0.989
Col 0h rep1 vs. Col 0h rep3	0.984
Col 0h rep2 vs. Col 0h rep3	0.972
Col 0.5h rep1 vs. Col 0.5h rep2	0.987
Col 0.5h rep1 vs. Col 0.5h rep3	0.991
Col 0.5h rep2 vs. Col 0.5h rep3	0.978
Col 1h rep1 vs. Col 1h rep2	0.905
Col 1h rep1 vs. Col 1h rep3	0.996
Col 1h rep2 vs. Col 1h rep3	0.923
Col 2h rep1 vs. Col 2h rep2	0.983
Col 2h rep1 vs. Col 2h rep3	0.965
Col 2h rep2 vs. Col 2h rep3	0.982
Col 4h rep1 vs. Col 4h rep2	0.914
Col 4h rep1 vs. Col 4h rep3	0.984
Col 4h rep2 vs. Col 4h rep3	0.968
Col 8h rep1 vs. Col 8h rep2	0.991
Col 8h rep1 vs. Col 8h rep3	0.983
Col 8h rep2 vs. Col 8h rep3	0.987

**mRNA-seq**	
Col rep1 vs. Col rep2	0.990
mut rep1 vs. *mut* rep2	0.991
**Ribo-seq**	
Col rep1 vs. Col rep2	0.993
mut rep1 vs. *mut* rep2	0.994

Table S9. Summary of the ribo-seq reads.

Library	Total reads	Filtered reads [a]	Uniquely mapped reads [b]
***mut*-rep1**	78412994	12432546	5855626
***mut*-rep2**	71967900	12521255	6144362
**Col-rep1**	66296086	11584080	5052239
**Col-rep2**	74821007	13537542	6411979

[a] After the PCR duplicates were reduced to 1 and adaptor sequences were trimmed, the number of reads with ≥18 nt in length. [b] Number of reads uniquely mapped to the *Arabidopsis* genome (TAIR10).

Table S10. Summary of the mRNA-seq reads.

Library	Total reads	Clean reads [a]	Mapped reads [b]	Uniquely mapped reads [c]
***mut*-rep1**	6869587	6861373	6276264	5710129
***mut*-rep2**	8235896	8227352	7467765	6733620
**Col-rep1**	7779688	7773872	7278976	6492884
**Col-rep2**	6223549	6217652	5650681	5115968

[a] Number of reads with an average Phred quality score greater than 20. [b] Number of reads mapped to the *Arabidopsis* genome (TAIR10). [c] Number of uniquely mapped reads.

Table S11. List of primer sequences.

ID	Description	Sequence (5′–3′)
**For genotyping *atpab* mutants (Additional file 1: Figure S2a)**		
HX860	*atpab4-1* forward	GGTTATGTCAACTACAGCAACAC
HX861	*atpab4-1* reverse	CAAAAGTATGAGGTTCCGATC
cx7662	*atpab8-1* forward	ATCAACAACAGCTTGTACCGG
cx7663	*atpab8-1* reverse	CAGTCTGTTTTCGGAAGCAAG
cx7672	*atpab2-1* forward	ATTCGAAAGTGTCAAACACGC
cx7673	*atpab2-1* reverse	TAATAAAAATGTTGCCAGCGC
**For *AtPAB* RT-PCR for full length coding sequences (Additional file 1: Figure S2b)**		
cx7554	*AtPAB2* forward	CACC **ATG**GCGCAGGTTCAACTTCAG
cx7555	*AtPAB2* reverse	**TTA**AGAGAGGTTCAAGGAAGCG
cx7556	*AtPAB4* forward	CACC **ATG**GCTCAGGTTCAAGCTCCTTC
cx7557	*AtPAB4* reverse	**TCA**TAAATGATCATTGATGG
cx7558	*AtPAB8* forward	CACC **ATG**GCTCAGATTCAGCATCAGGG
cx7559	*AtPAB8* reverse	**TCA**AGGTACGATGTTGTCTCCAAG
**For *AtPABs* complementation vectors construction (Additional file 1: Figure S3a)**		

cx8366	*AtPAB2*-up [a] forward	GAATTC TATGCTTCCAATAGTGGATGATAGC
cx8367	*AtPAB2*-up reverse	CCATGG AGAGAGGTTCAAGGAAGCGAGC
cx8368	*AtPAB2*-down [b] forward	CTGCAG TAAATTGCTTTTTATCATTTGATC
cx8369	*AtPAB2*-down reverse	CTGCAG GTTCATGACGCAATGTACTGG
cx8370	*AtPAB4*-up forward	GGATCC AATCTCTACACAATGTCCAC
cx8371	*AtPAB4*-up reverse	CCATGG TAAATGATCATTGATGGAAAGTG
cx8372	*AtPAB4*-down forward	CTGCAG TGAGAAGCTTTTGTTCGAG
cx8373	*AtPAB4*-down reverse	CTGCAG GGGTCTGCATCCAACAATTTTTG
cx8374	*AtPAB8*-up forward	GGATCC GAAAGAAAATTGAACTTGGAAAG
cx8375	*AtPAB8*-up reverse	CCATGG AGGTACGATGTTGTCTCCAAG
cx8376	*AtPAB8*-down forward	CTGCAG TGAGAGTTCTAAGTTATATCTG
cx8377	*AtPAB8*-down reverse	CTGCAG TAGGAGACAACGAAAGCT
**For amplifying Northern probes (Additional file 1: Figure S3b)**		
cx7700	*AtPAB2* (+ cx7554)	TTCTGGGCTCTACCAACATACCACTC
cx7701	*AtPAB4* (+ cx7556)	CAAAACACCTGGATTTCCCATCTC
cx7702	*AtPAB8* (+ cx7558)	CCAAAGCCTTTAGACTTTCCTTC
cx461	*FLC* forward	CGC GGATCC ATGGGAAGAAAAAAACTAGA
cx462	*FLC* reverse	CCG GAATTC CTAATTAAGTAGTGGGAGAGT
**For RIP-RT-PCR (Fig. 2e, g and Additional file 1: Figure S6)**		
At-5	AT3G14940.1 forward	CAGGAGCTAGATGAGTCTCTG
At-6	AT3G14940.1 reverse	CTCGGATTACCATCACGATCAC
At-7	AT5G12860.2 forward	CAGTCTCGTCTTCAGTGAAGC
At-8	AT5G12860.2 reverse	CCAATCGTCTGCTTAATCGTG
At-9	AT1G12110.1 forward	GTACAAGACGATGTTGGACGC
At-10	AT1G12110.1 reverse	GAACGGAATTGTTCAGTGTGTG
At-11	AT4G40090.1 forward	ATGGCTCTTAAGACATTGCAAG
At-12	AT4G40090.1 reverse	TTGGTGGAGTTGTTGGAACC
At-13	AT1G30360.1 forward	GAGCTAATCAACGAGTCTGTAG
At-14	AT1G30360.1 reverse	GAATGGTCACTGCAACAAAGAAG
At-15	AT4G27260.1 forward	CTCTGACTCTTACCAGAGCATG
At-16	AT4G27260.1 reverse	CCAGGACGACTTCTTGCACTC
At-17	AT3G18890.1 forward	CATTGTTTGGTGGTCAGGTCTC
At-18	AT3G18890.1 reverse	GCTTCATCCTCTTTAGGAGCTG
At-23	AT1G77760.1 forward	CACGTGGAGTGTTTGTGAGCT
At-24	AT1G77760.1 reverse	CTATGAGGCTTGCACACGTTG
At-25	AT4G39910.1 forward	CTCGTCATCATCATCTCCCGAG
At-26	AT4G39910.1 reverse	GTGCTTCTTGTAAGCTGCAGC
At-29	AT1G71880.1 forward	CCGTTCACAATGACAAAAGCTTG
At-30	AT1G71880.1 reverse	GGAACCAAGCGATCCAGTTTAAG

[a] "up" represents the promoter sequence (2.0-kb region upstream of the transcription start site) and the genomic region between the transcription start site and the site right before the stop codon. [b] "down" represents sequences of 3′-UTR and the terminator (0.5-kb region downstream of the transcription end site).
